# Organizational Context and Healthcare Reforms: What Effect on the Professional Distress of Canadian Social Workers and Social Service Provision?

**DOI:** 10.3389/fsoc.2021.651240

**Published:** 2021-10-12

**Authors:** Maude Lévesque, Lilian Negura

**Affiliations:** University of Ottawa, Faculty of Social Sciences, School of Social Work, Ottawa, ON, Canada

**Keywords:** healthcare reforms, new public management, organisational constraints, professional distress, clinical social work

## Abstract

This study examined the lived experience of Canadian clinical social workers in light of the organizational context in which they work. The literature indicates an alarming rise of occupational psychological distress in social workers, which aligns with the rise of the neoliberal ideology within the Canadian healthcare sector. While we know that organizational constraints and structural reforms affect social worker’s workplace well-being, it remains unclear how these changes are represented by front-line practitioners and how they affect the provision of social services in healthcare settings. To deepen our understanding of this issue, we conducted a thematic analysis of thirty semi-directed interviews with social workers currently practicing in three Canadian cities (Ottawa, Moncton and Winnipeg). Discussions of daily work life, responsibilities, autonomy and subjective understandings of the social worker’s role revealed which organizational constraints were the most significant in everyday practice and how they relate to their professional identity and mandate. Provincial healthcare reforms were generally found to have negative effects on clinical social workers, whose struggles for recognition were impaired by the fundamentally neoliberal ideologies behind the large-scale restructuring of service provision, themselves at odds with the humanistic principles of social work. Our findings further suggest that structural changes under the New Public Management frame could be detrimental to both the quality of services provided by clinical social workers and their well-being. Overall, this investigation highlights the importance of organizational improvements in the workplace through systemic changes that would concurrently target managerial expectations, resources allocation, autonomy, work-life balance and respect for professional values.

## Introduction

### Healthcare Reforms and Social Work: A Tumultuous Affair

Canadian healthcare operates as a single-payer system with universal coverage (Ivers et al., 2018). Since its introduction in the late 1960s, provinces have been tasked with its specific administration and delivery, provided they fulfill the general mandates set forth by the *Canada Health Act*. Thus, while acute medical needs and emergency care have remained accessible for individuals of all income brackets nation-wide, healthcare management has been the subject of numerous reforms over the past decades. These reforms have all kept with the general tendencies of the New Public Management (NPM) (Hutchison et al., 2011). Briefly, in the healthcare sector, NPM equates the management of public services with the management of services in the free market and seeks the optimization of services primarily through an efficient use of economic resources and consumer-centered interventions. As such, it promotes a result-oriented governance for both patients and taxpayers (Sebai and Yatim, 2018).

This philosophy is ever present in the newly established Ontario Health agency, which, in 2019, overtook singular organizations focused on distinct healthcare needs and services (i.e. the former Cancer Care Ontario or the Ontario Telemedicine Network) to streamline government spending and “ensur[e] financial accountability” (p.1) in every branch of medical practice (Government of Ontario, 2021). Cost-efficiency is here presented as a central concern, entrenched in heightened digital monitoring and supervision, in itself a pillar of NPM practices (Abramovitz and Zelnick, 2018). The latter’s ubiquity across the national healthcare landscape is evidenced by the striking similarities found in the recently implemented Shared Health authority in Manitoba along with the well-established, yet similarly inclined, New Brunswick Health Council. Indeed, both province’s healthcare authorities operate under patient-centered, cost reducing models of service delivery guided by principles of care standardization, performance measurement and increased accountability (Government of Manitoba, 2021; (New Brunswick Health Council. 2016).

As result, within every reform of the past decades, public expenditures were restricted with the highest scrutiny. Taking Ontario as a recent example, the 2019 provincial budget introduced a decrease of the Ontario’s Children, Community and Social Services Ministry’s funds by 1 billion dollars over 3 years, citing “cost-efficiency” priorities (Monsebraaten, 2019). A ubiquitous reality across all public services, this austerity has led healthcare institutions to foster business-like order, expecting evermore quality, efficiency, professionalism and overall devotion from their employees (Bart and Hupfer, 2004). The computerization of the workplace has paved the way for the constant monitoring of front-line practitioners, whose work hours are increasingly devoted to filing reports, work notes and minutes for every act performed in what is now called the “bureaucratization of social work” (Burton and van den Broek, 2009). These scrupulous records are a key factor in obtaining funding for community organizations and public institutions alike. The required constant amelioration in service is paired with the will to optimize expenses, so as not to indulge in frivolous public spending. Under such scrutiny, social workers have to grapple with increasing austerity and licensure dubiously rationalized in terms of deficit control (Baines, 2004). To ensure the renewal of funding, institutions are thus forced to increase the demands addressed to their employees without additional resources.

The performance pressures arising from this acute monitoring have not necessarily resulted in better primary care. As the unit of measure of performance is that of reports, bureaucracy has been placed at the forefront of work priorities, even superseding patient/client care itself (Burton and Van den Broek, 2009; Gray et al., 2015). This shifting emphasis has been particularly challenging for social workers, whose ethics compel intervention and client interaction above managerial duties (Burton and Van den Broek, 2009). Social workers in a healthcare setting have also been requested to reframe their professional role from a value-based setting (deemed inefficient) to standardized practices (mainly evidence-based), which are better suited to broad monitoring and institutional integration (Walshe and Rundall, 2001). These NPM-informed policies have further entailed profound ethical dilemmas for clinical social workers faced with increasing health inequities. Nova Scotia, for instance, saw the sudden monetization of hospital beds taken by elderly patients awaiting a nursing home transfer whose unique cost had to be charged directly to patients by social workers (Weinberg and Banks, 2019). Some clinics in Ontario, for their part, started establishing a service cap for clients with mental health social workers being required to dismiss their patients after a maximum of six meetings regardless of whether they had been referred elsewhere or were still in need of services (*Ibid*.). These examples offer but a glimpse of the profound transformations imposed on social work practice over decades of NPM reforms.

The gradual erosion of humanistic principles through organizational constraints has been difficult for social workers and is strongly associated with rising tensions and workplace dissatisfaction (Burton and Van den Broek, 2009). Although significant, the complexity of this association has not been fully explored. The most predominant reported effect of organizational constraints for this occupational group concerns the transformation of the mandate of social workers to a regulatory-based practice, thus prompting value conflicts and moral distress (Jessen, 2015). A limited autonomy in practice, the difficult conciliation of professional and personal lives and the tying of funds to monitored services have also been shown to negatively impact the work experience of front-line practitioners (Ben-Zur and Michael, 2007; Bouterfas et al., 2016; Kim and Stoner, 2008). This comes in addition to the fact that social work is known to be amongst the professions with the highest rates of burnout and work-related stress (Wulsin et al., 2014; Evans et al., 2005). Yet, the questions of how and under which conditions organizational constraints affect social worker’s well-being and the provision of social services, although crucial, has seen little empirical exploration under the lens of social experience and professional representation. Rather, workplace stress theory dominates the broader inquiries about healthcare professionals as a whole.

### Social Representation Theory and the Workplace Experience: Toward a Deeper Understanding of Professional Life

We know from the founding figures of the theory of professional stress, Karasek and Theorell (1990) and later Siegrist (1998), that autonomy, social support, higher demands and discrepancies between effort and reward are determinants of workplace well-being. In the context of neoliberal reforms, healthcare professional’s occupational welfare is primarily threatened by two overarching factors of professional distress (Lornudd et al., 2015; Vézina and St-Arnaud, 2011): a deficit in managerial support (Blackstock et al., 2015) and a reduced job control (discretionary autonomy) (Wilson, 2015; Day et al., 2017). While highly relevant, the available literature offers limited insight into the lived experience of healthcare social workers outside individual determinants of well-being. A qualitative inquiry is further required in light of the organizational changes observed in healthcare institutions following the advent of NPM.

We turn to the theory of social representations (Moscovici, 2001) in order to consider the complex subjectivities inherent to the front-line practitioner’s shared self-perception. Occupational stress models, while useful, often fail to account the professional lives of healthcare social workers as a matter of shared subjective experience (see Graham and Shier, 2014 for how expectations of social workers affect their workplace well-being). Under the theory of social representations, the lived experience of clinical social workers is understood as a collective significant meaning assigned to a given situation within their workplace. This subjective emotionally charged response is shaped by the group’s pre-existing social representation. A social representation can be defined as “a component of lay knowledge, a theory of common sense on an object of significance to a social group, produced by natural logic and social thought” (Negura, 2016: p.12). Indeed, where a lived experience pertains to what is jointly felt in relation to a given situation, a social representation guides the way in which the group reacts to this situation by giving it meaning. The social representation itself is co-constructed by way of objectification and anchoring; processes of thought mobilized together to first, by objectivation, make concrete an abstract object and to second, by anchoring, situate said object within the group’s pre-existing thought system (Rateau and Monaco, 2013). Social representation can inform the professional experience of healthcare social workers by participating in the construction of meaning in their occupational lives. Every representational element comprised by the lived experience of healthcare social workers will thus be explored in the context of the organizational workplace changes brought on by the NPM. Additionally, delving into the processes of objectification and anchoring at play in such lived experience will strengthen our understanding of how the social representation of the practice operates under the pressures of NPM-reforms as part of the clinical social workers’ ongoing negotiation of their lived experience.

When contextualized to the workplace, a social representation can take the specific form of a professional representation. As such, it is defined as a thought-system on a given social object co-constructed by a professional group (Bataille, 2000; Moscovici, 1976). It contrasts with the social representation of non-professionals (here managers and patients) which lack the shared social-work professional practices and formal training at the heart of the professional representation’s edification. As presented by Bataille et al., (1997), a professional representation is distinct as it comes to define the social identity of professionals. As such, to understand how organizational constraints impact the professional lives of social workers, it is not sufficient to look only at what policies and protocols have been subjected to change; one also needs to understand how these workplace transformations have been received, interpreted and integrated by the professional group they impact (Bataille et al., 1997). Mobilizing a social representation as a gateway to lived experience allows us to uncover the ways in which healthcare reforms in three Canadian provinces (Ontario, Manitoba and New Brunswick) have transformed the workplace in health care institutions, how the ideologies they feature have been integrated or resisted by front-line practitioners and how, finally, they may have influenced the professional distress of social workers and the delivery of social services to patients. These avenues of inquiry thus inform our general research question: how is the professional experience of social workers influenced by the current organizational constraints of healthcare settings?

## Materials and Methods

Thirty participants were retained for our sample from three large Canadian cities, each from a different province: Moncton (New Brunswick), Ottawa (Ontario) and Winnipeg (Manitoba). Our participants all shared four key characteristics: being francophone, female, a registered social worker and practicing in a healthcare setting. The interviewed social workers were equally sampled from every city (see Table 1).

**TABLE 1:** Distribution of the sample (*n* = 30).

Sociodemographic characteristics		(*n*)
Age	20–25	1
	26–35	15
	36–45	10
	46–55	2
	56–65	2
Municipality	Moncton	10
	Ottawa	10
	Winnipeg	10
Years in current position	0–5	15
	6–10	11
	11–15	4
Workplace setting	Institution	25
	Community	5
Employment situation	Part-time, determined duration	0
	Part-time, undetermined duration	3
	Full-time, determined duration	4
	Full-time, undetermined duration	23

The gendered and linguistic demographic restrictions of our sample were elected to better explore specific nuances in the experience of healthcare social workers. Based on our earlier studies (Lévesque et al., 2019), the minority linguistic affiliation was generally expected to influence the experience of daily life (whether in terms of professional standing, added tasks or stigma). We also posited the possible link between a minority linguistic affiliation and the experience of professional distress in a healthcare setting as this population’s social capital is lower than its anglophone counterpart (Bouchard et al., 2006) and the perceived health status of individuals differs according to linguistic groups (Bouchard et al., 2009). Finally, we restricted our sample to a self-identifying female demographic to better account for the gendered reality of the profession, with 83% of social workers being women (Statistics Canada, 2019). As the second article to a series of four on the professional distress of clinical social workers (Lévesque et al., 2019; Lévesque et Negura, 2021; Negura and Lévesque, 2021), this study features a sample with particular eligibility criteria set for participation. Indeed, the members of our sample were initially invited to partake in a broader study on the professional distress of social workers. They had to self-identify as having personally experienced professional distress, a concept defined by the research team in our interview guide as “a notion used to encompass the many forms taken by psychological distress at work, including stress, depression, professional burnout, suffering, etc.”. Beyond our specific interests, the methodological implications of these criteria are explored in the limitation section of our paper.

The recruitment was conducted on a voluntary basis without the incentive of remuneration between April 2017 and August 2018. It was carried out through three distinct methods: by word of mouth, through posters in healthcare establishments (both institutional and community-based) and through meetings with teams of social workers in healthcare settings. Our participants were offered the choice of a convenient location for the interviews, leading many to opt for either public coffee shops or private spaces at their own workplace. Pseudonyms were either chosen by or randomly assigned to participants to protect their privacy. The research was reviewed by the University of Ottawa’s ethics committee and received its full approval.

### Data Collection

Our data was collected through semi-structured interviews conducted by three research assistants, each assigned to their own city. The data collection efforts were part of a larger study whose general purpose was to better understand professional distress in social workers. As such, our interview guide touched upon three larger themes which are each thought to play a part in the professional distress of social workers: 1) professional identity, 2) education and professional experience through organizational constraints and 3) the specific manifestation of professional distress. For the purpose of this paper, only the verbatims related to the second theme were retained for analysis in order to focus on the shared experience of healthcare social workers’ in reformed settings. The education and professional experience theme presented participants with questions about the workplace experience, their organizational constraints and the value conflicts arising from disparities between academic and their field experience. The transcriptions of the interviews were coded by the main researcher within the Nvivo 11 platform, which organizes the qualitative data and facilitates the ensuing analyses.

### Data Analysis

This study aims to develop our understanding of the lived experience of social workers in a healthcare setting following the past decade of healthcare reforms. Towards this goal, we sought to explore the elements of the professional representation that inform the shared experience of healthcare social workers in their organizational context. To achieve this, a thematic content analysis was performed on the relevant sections of interview transcriptions. We began by examining the particular workplace situations that our participants associated with NPM-informed institutional changes. With such specific cases in mind, we were able to investigate how the clinical social workers from our three sampled provinces felt, or, in other words, the nature of their lived experience. By delving into their explanations for these reactions, we ultimately unearthed the group’s social representation of their practice. At the core of persisting occupational tensions, the representational elements uncovered allow us to debate the impact of NPM praxes at a fundamental level of professional functioning.

Broadly, our thematic analysis comprises three large processes established by Miles and Huberman (1994): the reduction of the data based on the focus of the research, its organization and its verification. After an initial identification of the elements at play, the significance of the uncovered themes was discussed by both authors of the article on the basis of recurrence and coherency among participants. The NVivo 11 platform facilitated the inductive exploration of the content to be further structured by a quantitative measure of frequencies. The frequencies of the uncovered representational elements were calculated in two manners. The number of participants evoking a given element of their workplace experience was interpreted as a sign of its prevalence, while the number of times this element was mentioned within all interviews was an indication of its importance for social workers (Negura, 2006). Every calculation was corroborated with the thematic analysis of content to validate the assertions of significance about a given representational element.

This analytical method has been shown to be particularly effective to explore the inner dynamics of representational content (Dany, 2016; Negura, 2006) and has been previously established as an effective technique in social representation’s studies to explore the more complex subjectivities of shared professional knowledge (Morant, 2006). The use of a basic statistical description as a validating technique allowed us to apprehend the representational ties between the issues at play for the professionals themselves while also providing us with a manner of seizing the strength of the representational components related to their shared experience. It follows that each of these elements was debated not only in terms of its subjective significance but also with regards to its popularity among social workers and its relative importance for them.

## Results

Matters of daily professional life for social workers in a healthcare setting were analyzed according to both the concrete practices (i.e., interventions, protocols) they involve and the subjective interpretations (i.e., mandates, expectations, values) they generate. Two encompassing components emerged from our data that effectively structure the lived experience of healthcare social workers: cumulative organizational constraints and a paradoxical autonomy. For each theme, distinct situations generated by shifts in institutional policies shape the lived experience of our participants. Herein, we argue that the latter’s feelings of inequity, uncertainty and frustration among others arise from tensions with the professional representation that they rely on to function as an occupational group (Hyslop, 2018). As such, each theme and the representational elements that they subsume bears implications for front-line practitioner’s well-being, the integrity of their professional identity, and likelihood of experiencing professional distress. Let us begin by exploring what constitutes cumulative organizational constraints for healthcare social workers and the meanings attributed to these constraints.

### Cumulative Organizational Constraints

The significance of cumulative organizational constraints on the healthcare social workers’ occupational reality is unequivocal. A unanimous mention across every participant, a lack of time and resources, an excessive workload and confusing guidelines all compound the stress associated with an emotionally charged profession. These managerial limitations not only influenced the clinical social workers in practice, but also through the meanings that these limitations elicited, they proved overall detrimental to our participants’ subjective professional experience.

### The Trinity of Hardships: Time, Resources and Workload

Lack of adequate time, the need for additional resources and a disproportionate workload represent by far the most prevalent constraints deemed to worsen the workplace experience of social workers, comprising half of all occurrences pertaining to organizational constraints (49.18%). Their interrelation is indisputable, as with more time available, the caseload could be better managed, and with adequate resources workloads could be completed more efficiently or delegated, freeing up more time or reducing the number of tasks, etc.

Of the three elements, the work overload was sourced from the largest number of interviews (*n* = 25) and referenced most often (50 times). The excess work imposed by organizational constraints was found to have a variety of impacts, both emotional and on their ability to perform the tasks at hand. For one, it negatively affects the social workers’ feeling of professional competency:

[..] I felt alone, stuck with a heavy case. The entire interprofessional team expected me to know what to do and I didn’t know… I didn’t know what to do. That was the beginning, then once it [the work] started overflowing in one case, it had an impact on all of my other cases… I didn’t feel competent as a social worker. – Emma

This erosion of practitioners’ self-assurance is further exacerbated by the very nature of the work, here objectified as crisis management. With most front-line practitioners describing their mandate as “putting out fires” and dealing with constant crises, many had difficulty coming to terms with not offering the full extent of services they could have provided if given appropriate time and resources:

We have too much to do with too little time, so we have to cut our work in two or three because I can’t pursue step parenting with families. Yes, I have some time to meet with them but definitely not to sit down and complete psychological assessments. Lacking time impacts the quality of my work. – Bernadette

Participants emphasised the difficulty of the work itself, revealing, in essence, how work cannot be solely measured in terms of time spent in an office. Work overload is thus not only a matter of hours in a day, but of the types of tasks performed:

A young girl screamed at the top of her lungs for 45 min without pause […] She was in a complete crisis, with screams, screams and more screams. I couldn’t hear myself speak and I didn’t know what I was doing anymore, I needed help and couldn’t calm her down over the phone. After 45 minutes of that on the phone, it’s exhausting. – Monique

This issue of overload was found to be difficult to explain to the higher administration, which only pay attention to the statistics made available to them. In essence, this serves to anchor the negative emotions stemming from unrepresentative performance metrics to the representation of the practice. Indeed, when documentation prevails over patient care, the priority shifts, incurring much frustration:

We have to do more with less. We have more constraints with statistics, documentation and we can’t always be with the patient or their family. – Sasha

The mandated paperwork was described as ever growing, expropriating services that should have been directed to clients. More so, the constant notetaking was presented as distinct from the professional practice itself, less a matter of social work than an institutional imperative:

For every hour that I spend with my client, I can’t tell you how many hours I have in paperwork afterwards. – Maria

*It’s clear that after working [as a social worker] for 7* *years, it’s not quite the same idea [of the profession] that I had in the beginning. It’s more so paperwork versus social work, so yeah, it’s different. It would estimate that 30% [of my work] is family intervention and the remaining 70% is paperwork.* – *Jordana*

The prioritization of documentation, while counter to the preferences of social workers, is however described as an unavoidable requirement. In effect, it serves as the last line of defense against budget cuts said to threaten front-line practitioners’ job security:

The supervisor I had before could see the work that we needed to do and the volume of cases we had so she really pushed for more workers and we got them. She conducted studies, research, she had basically written a little booklet to show the numbers to prove that we needed people […] – Jordana

Social workers have to shoulder the dual responsibility of providing services to clients while concurrently advocating for their jobs to their own employers. When dismissals occur, this decrease in resources perpetuates the worsening of the workload as a rapidly decreasing number of social workers have to take on their former colleague’s responsibilities:

I: […] In reality, there should be two, maybe three people for the work that I do. If we go back 15 years ago, there were three social workers that did my job for 26 clients. Now I am alone.

R: Do You Know why It Changed?

I: Budget Cuts – Héloïse

Legitimately, the growing frustration felt by our participants pertains to recognition. Budget cuts are seen as evidence that decision-makers misunderstand their profession. This produces a tension between these managerial practices and their professional representation, a representational dynamic anchored in the humanitarian values they hold firm. In a context where clinical social workers are already stretched thin, cuts made to their team can only be explained in terms of this undervaluation of their professional contribution and mission:

You know, I am pretty sure that if you gave them [politicians and other government authorities] only the clothes they have on their back and left them under an overpass, they wouldn’t cut our jobs quite as fast, you know. They have no idea what the reality of our patients is or even that of their employees for that matter. – Héloïse

The mistrust in the administration is heightened not only by the implementation of social policies or budgets cuts but also by the management of tasks in the workplace. The way in which the work is assigned was described as deceitful and engendering uneasiness in front-line practitioners, who viewed management as purposefully creating caseload excess:

[…] It was really horrible, I was told I would start little by little with 4 casefiles and then, you know, I would suddenly have 5, then 6. Well I was thrown to the wolves as by the end of my first month. I had 33 as a brand-new social worker coming out of university […] - Jordana

Social workers are thus left to their own devices to deal with excessive workloads, as well as lack of resources and a justified sense of insufficient time. These constraints, in turn, raise common issues around one’s sense of competency, trust and feelings of safety. Further difficulties are revealed when breaching the topic of supervision and workplace guidance, where standards have changed in accordance with managerial reforms.

#### Conflicts of Values and Priorities: The Managers and Social Workers’ Deontological Tug of War

Tensions between clinical social workers and their management emerged in the discourse as another more immediately observable representational element which shapes the lived experience of social workers. As for the previous elements pertaining to organizational constraints (the lack of adequate time, the need for additional resources and a disproportionate workload), it simultaneously affects the font-line practitioners’ practices while influencing the shared meaning given to their experience. To start, it perpetuates a state of confusion around the autonomous management of workloads since, for more than a third of our sample, expectations and guidelines were not immediately apparent.

Some attest to having a general impression of, or “a feel” for, what managers expect, but without receiving any specific instructions for the work itself:

I: What are the expectations that management has for your work?

R: Hum, for us to have respect, I think to respect patients, to work in teams and to encourage a good synergy. That’s about it, nothing concerning our work at all. – Philomène

As standards of practice and guidelines are not readily available to counsel social workers on their duties, it leaves the team of front-line practitioners scattered along a spectrum of practices, standards and priorities:

So, what are the instructions for our work, well…there is nothing written. For example, we need to make case notes and conduct interventions, but we have nothing that tells us for how long it should be done. And for the notes that we use to describe our interventions, we do them all differently. – Michèle

This tension manifests itself primarily in terms of a discrepancy between the representation of care, an important aspect of the professional representation of social workers, and that of management, which is explained, among other things, by the lack of clinical social workers in management positions:

Ah, expectations… well it’s a different perspective, you know, the medical one. For us, we have a different perspective on things […] it’s a bit hard, they share a different line of thought. – Fesefel

This divergence in perspectives creates tensions among the social workers’ own deontology, their formal training and their employer. While not specific, the administration’s expectations rarely align with the values of social work:

[…] we struggle to be able to meet the demands of our employer while being faithful to our role, our training or simply to how we see ourselves as social workers. – Monique

Like working in a hospital, sometimes they don’t have the same vision as we do so they push us to do certain things that contradict our mandate, so we end up playing tug-of-war. – Bernadette

Unarticulated guidelines, while not a source of tension in themselves, are paired with an absence of the clinical support deemed imperative to practice in a healthcare setting, as reported by 23 participants:

Another constraint is that we lack clinical supervision. That’s really lacking. Yes we help each other, but sometimes it’s really above our abilities, you know, narcissistic personality disorders and stuff like that, we don’t all have training for these things, like for me it’s just not my expertise at all. – Maria

The additional professional training sought after by front-line practitioners is not an available alternative for supervision, as funding is in short supply:

The lack of training comes from the lack of funding. You know the budget this year is 200 dollars or something per social worker for training. 200 dollars, you know, it makes no sense. – Maria

The absence of supervisors incurs further unexpected burdens on social workers, who depend on each other for both pragmatic and emotional support:

First, we would need clinical supervision because that’s something we don’t have. We have a supervisor, but he isn’t a clinical supervisor as I said because he has no idea what our job is […] there’s always a lot of staff turnover, so the personnel have very little experience, and when I experience something difficult I feel bad to go see them because they can’t help. Most of the time the opposite happens, and they come to vent to me, so on top of having my clients, I have all the team to take care of. – Monique

Overworked and pressured on all fronts, social workers are compelled to sacrifice their own rights to complete their duties within a workday:

We just can’t, sometimes we just don’t take breaks, we skip lunch at times. – Fesefel

They must further display great self-reliance to practice with very limited resources, with some social workers resigning themselves to the fact that they will be working with perpetual deficits:

I try not to think about it [lack of resources]; I don’t want it to affect my daily life […] I work with what I have, with the resources available. Becoming frustrated by the lack of resources is just going to exhaust you and nothing positive will come out of it. So, you just have to accept it and work with what you have. - Lillian

The greatest risk to well-being emerges when drastic coping mechanisms are employed to manage competing expectations and overbearing constraints in an attempt to correspond to the professional representation (Lévesque et al., 2019), resulting in the shattering of boundaries between professional and personal lives reported by 18 distinct sources. In the following excerpt, our participant shares an such an instance of compensatory overworking immediately prior to her burnout:

I went beyond my work. I went to the store. I purchased her clothes, underwear and make-up because the lady told me that, before, she would never leave without her lipstick. I bought everything from my own pocket. I also went to my grand-mother’s wardrobe to take the clothes that she wasn’t wearing anymore. I washed them all and brought them to the patient for her to have something, a little something of her own, you know. – Sophie

While genuine devotion is consistent with the social worker’s ethos, no mechanisms are put in place to ensure balance and relieve front-line practitioners when they voice a need for some time off, leaving them arguably chained to their responsibilities at all times:

When you miss a day at work for a family emergency, or you decide to go on vacation or whatever the reason is for you to miss a day, you know you’ll be paying for it. You know that day that you want off isn’t worth it because you’ll be so overwhelmed when you come back it doesn’t make sense. – Héloïse

### Paradoxical Autonomy

Autonomy was found to be the most significant characteristic of the workplace, after organizational constraints. Mentioned by 17 participants and referenced 19 times, close to two-thirds of our sample discussed it in terms of its paradoxical nature. Paradoxical autonomy encompasses three representational elements of the workplace that together frame the shared experience of healthcare social workers and anchor it to their representation of the practice: the *value-based representation of professionalism*, the *exposure to litigation* and the *primacy of field experience*. When combined, these elements all point to the lack of guidance identified earlier as a constraint. Together, they reflect the representational tensions brought on by the clinical social workers’ unmet need for self-reliance.

#### Value-Based Representation of Professionalism

Let us begin with the *value-based representation of professionalism*, which was referenced by slightly less than a third of our participants. It is a matter of consensus that social workers require professional training to mobilise concrete techniques in order to fulfill their mandate:

If you don’t know how to intervene, if you require specific approaches, then you need social workers. There are just cases that can’t be dealt with normally. That’s why you need to have the techniques of a social worker, you need to be a social worker to intervene in these cases. – Letitia

For some participants, professionalism does not necessarily involve specific techniques but rather is described broadly in terms of a general attitude, tying into the representation of social work itself (Lévesque et al., 2019):

Professionalism, that’s important. I would say it implies neutrality, you know, impartiality I should say. I mean, the behaviour you must have, it’s to be always listening, amicable, smiling, empathetic, patient. Very, very patient. – Sophie

Our participants’ insistence on professionalism can be fully understood only when contextualized by the other defining characteristics of their workplace experience. This brings us to the *exposure to litigation*, a matter referenced 15 times by the 8 sources that brought it to our attention. The innate sense of professionalism, or possessing the correct behaviour in difficult work scenarios, is vital in light of the constant threat of lawsuits faced by social workers. As they perform difficult tasks, manage delicate information and are at the mercy of contradictory expectations from an allegedly distant administration, social workers are often the first targets of offended clients:

Since high-ranking managers don’t know what we do, they impose tons of things on us without consulting our professional Order. They will impose stuff that doesn’t make sense, and if there is a lawsuit, they aren’t involved, they aren’t even named! Last summer my manager ordered me to send out pictures of a patient who had run away which I couldn’t do because of confidentiality reasons and I got reprimanded for that. I thought it didn’t make any sense! – Philomene

The constant threat of litigation weighs heavy on social workers, not only due to its long-term implications, but also by how it undermines their very role as professionals and endangers their shared identity:

I could only see the negatives [when talking about her work]. You know, I would question myself on why I even became a social worker; if I’m just going to get in trouble or have my judgment questioned or be questioned with regards to my obligations towards the law. Is it really worth it to be a social worker? It’s the risk that comes with lawsuits, you know. – Lillian

This is particularly troublesome in the context of an autonomous practice, as protections are said to be limited for clinical social workers who provide services to a highly vulnerable clientele. These circumstances are aggravated by the reported unaccountability of administrators, who are described by social workers as distancing themselves at the sight of trouble and refusing to assume responsibilities:

It happens sometimes that you have a very difficult patient. When we can’t manage the patient, we go to our manager. “This is a difficult family, could you offer me some help?” […] She immediately throws the ball back at you “Well do this and that”. But I already did it. She throws the ball back again: “Well do this and that!” It feels like she’s distancing herself. And us, we are always the intermediary, so yeah, it’s tough! – Leti

The *primacy of field experience* is a vital aspect of this professionalism, as discussed by 12 sources, because the challenges of the work environment cannot be addressed through formal education alone. As attested by many social workers, nothing prepares them for the realities of work life:

In university you aren’t trained on how to protect yourself, never. So the first day on the job for me it was with child protective services and in my very first week I removed a child… no one prepares you for that. – Maria

First-hand experience is also crucial because it helps social workers to manage their role, which they describe as autonomous. In the scientific literature, discretionary autonomy is often identified as an empowering feature in the professional context (Papathanassoglou et al., 2012). In contrast, for our participants, autonomy was far from a positive aspect of their work experience due to the context in which it was granted. Indeed, an undue burden is felt by clinical social workers who describe navigating in an environment where their role boundaries are not consistently understood by their medical peers. As such, it is easy for interdisciplinary teams to delegate unrelated tasks to social workers, disturbing their work schedule and role expectations:

What happens generally is that I come in and organize my own agenda. But what ends up happening usually is that I am tasked with something else. We’re being called upon by nurses or chief-nurses or anybody. So a nurse that comes to you or a doctor says things like “Oh! Could you do this or that?” And the day never unfolds the way we planned it, it changes a lot. – Fesefel

In this way, their so-called autonomy permeates every aspect of the workplace experience. For one, it tends to aggravate the vulnerability of social workers to litigation because they are accountable for each decision they make:

*I eventually decided to place a call* [to child protective services]*. So, anyway, I think it was 6 months later, I was informed by the administration that the lady, her family, was prosecuting the hospital because they were my employer, because of me, because I had made the call and brought emotional harm to the family. – Lillian*

I think sometimes we are being made responsible for things that we haven’t instigated. Like, I’m not the one who caused the issue and yet, I’m the one who is responsible for problems caused by the administration, the ministry […] I find that very difficult. – Leti

Similarly, the *value-based representation of professionalism* and the *primacy of the field experience* only gain importance in the narratives of social workers due to the autonomy they possess. Indeed, as part of the social workers’ expected flexibility, every task is more cognitively taxing and requires the elaboration of adaptative strategies:

I take notes. I put everything on sticky notes in my agenda. And the sticky notes move around depending on what is happening. […] I do a lot of overtime. I manage crises before what’s less important. – Beatrice

Professionalism and the knowledge gained by field experience are thus key to help counterbalance the strain caused by this *paradoxical autonomy*. Participants with more years of experience were thus more at accustomed with the particular constraints of practice that they were faced with. This did not however alter the meanings attributed to their lived experience nor the rationale behind its inherent challenges, pointing to the enduring value-centered objectification of the social representation of clinical social work practice. Across participants, their given autonomy was hence not deemed fully authentic, as it is given without the appropriate tools to actualize the institution’s requirements:

The administration comes to us with a new initiative and says “make it work”. […] So with that it’s pretty much us deciding on our own tasks and sometimes we miss the boat on what to prioritize which creates stress in organizing everything. We don’t have an administrative office either […] so we do it all. - Alexa

**FIGURE 1:**
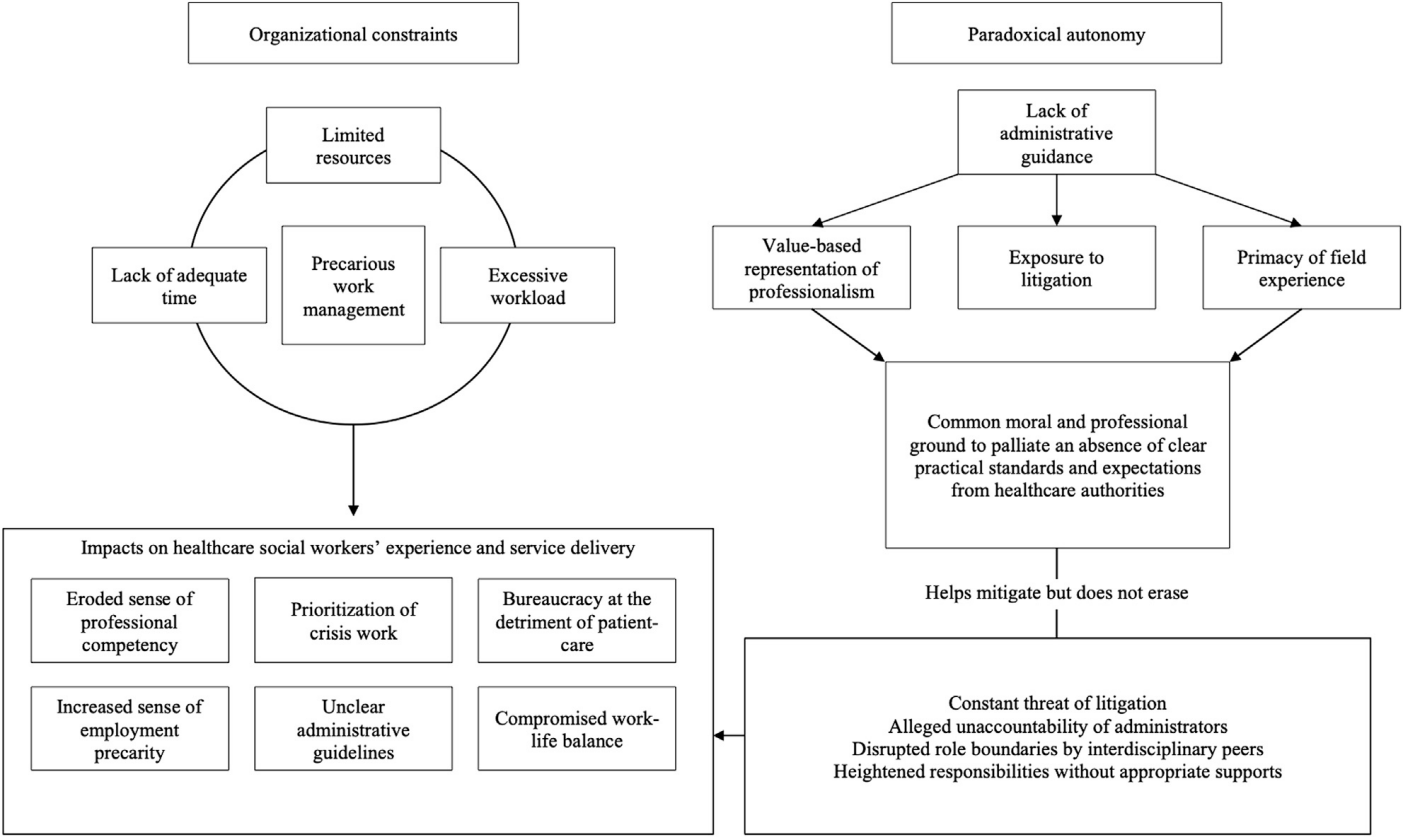
Representational structure of the lived experience of Canadian clinical social workers.

In summary, healthcare social workers experience unnecessary challenges, as they are made responsible for a professional practice without adequate resources or protections. They must further navigate confusing expectations and demands from their medical peers who unknowingly undermine the clinical social workers’ genuine professional values and norms. Autonomous practice in healthcare social workers is made conditional to the fulfillment of managerial priorities without the latter shouldering any meaningful legal accountability. Ultimately, autonomy without the appropriate support is denounced by our participants as negatively impacting their professional experience in a context where an arduous practice is complexified by limited resources, confusing expectations of service delivery, dehumanized care and ever-changing requirements from unpredictable clients and managers alike. For the sake of clarity, these findings are here summarized in a synoptic model of the representational structure of the lived experience of healthcare social workers (see Figure 1).

## Discussion

Our results attest to the decisive influence of our three provinces’ healthcare reforms on the professional experience of clinical social workers. The cumulative organizational constraints faced by front-line practitioners are perhaps the most tangible evidence of the problematic transformation of the workplace under a NPM philosophy. These external systemic challenges are further compounded by a paradoxical autonomy of practice, structured by its representational components of *value-based representation of professionalism*, *exposure to litigation* and *primacy of field experience*. Each is a necessary gateway to understanding the meanings that social workers assign to their lived experience of practice using these representational elements anchored in common professional values. Beyond the reach of this study, the shared occupational experience of healthcare social workers may help situate the systemic roots of their professional distress and its negative consequences on the quality of social services provided to patients. Indeed, our findings point to our clinical social workers’ workplace suffering arising from incompatibilities between the shared professional representation and the real practice they experience. Their professional representation resists change despite the pressures and the broader expectations of practice clinical social workers face within a neoliberal workplace climate. In our sample, the discrepancies between the professional representation of clinical social workers and the NPM-driven management in the employing institution led to many conflicts of practice while greatly complicating the exercise of the profession. These managerial procedures reveal not only a divergent neoliberal ideology and vision of care but also a different (mis)representation of social work. Let us explore, then, the characteristics of this lived experience, the processes that underline it and discuss what constructive avenues of change could practically address the challenges uncovered herein.

### The End of the Line: Social Workers as a Palliative Solution to a Flawed System

Our qualitative analysis uncovered how social workers must contend with four concrete barriers in the exercise of their functions: a deficit in resources, a limited time, an excessive amount of work and an unsupportive management. This quaternary of obstacles was the most prevalent aspect of the professionals’ narrative and points to multifaceted issues in the work life of social workers in a healthcare setting. These cumulative constraints all derive from gradual policy implementations in healthcare institutions driven by cost-efficiency, personal accountability and increased monitoring, indeed all indicative of NPM reforms (Hutchison et al., 2011). Our participants’ lived experience aligns with current empirical research as it is said to specifically entail notetaking after every professional act to assess output (Burton and van den Broek, 2009; McGivern and Ferlie, 2007), heightened employment precarity as the primary target of budgetary cuts (Baines, 2004), strict expectations of self-sufficiency (Page, 2005), limited managerial support and unavailable funding for continued education (Abramovitz and Zelnick, 2018). Understanding managerial practices through the discourse of clinical social workers under a common professional banner, made possible by our representational analysis, allows us to understand how the NPM reforms affect the *raison d'être* of this professional group and how their shared professional distress operates.

As presented in the results section, every organisational constraint and tension with administrators lead to similar consequences: the inability to complete the mandated tasks set by their representation of the profession and/or a decline in the quality of available services under the subjective standards of practice. The harm caused by these unsatisfactory outcomes is much more adverse than what is immediately observable. Indeed, the need to work in a constant state of urgency leaves long-term, more successful interventions aside to prioritize stress-inducing, high-pressure casework, in essence objectifying the social representation of the practice as one of crisis management. Since crisis management is perceived as a temporary situation par excellence, representational transformation is not possible. Indeed, as (Flament and Dri, 1994) has shown, for a representational change to take place, the members of a social group must perceive the new situation as irreversible and permanent. The objectification of the representation of practice as crisis management can be understood as a schema-strange (Guimelli and Rouquette, 1993), a perceived abnormal and exceptional element within the representational structure. This has an effect on the lived experience of social workers who feel they are not doing their job fully. The inability to practice holistically is felt as eroding the social workers’ sense of competency and purpose because they are limited to providing insufficient help. Social workers thus suffer from the tension between institutional work expectations and their professional representation of social work. The latter is anchored, as we have seen in our results, in a set of values considered central to the profession such as empowerment, respect or social justice (Lévesque et al., 2019). This anchoring takes place through education and professional socialization (Banks, 2012). The values in question are in turn at odds with the principles of NPM.

These findings could help explain the retention problem observed amidst the profession (LGA, 2009; Kim and Stoner, 2008) beyond the simple difficulty of the job. Put simply, the gratification of clinical social workers is compromised by the rushed casework of the institutional environment, thereby limiting their job’s rewards while heightening its demand. The resulting strain felt by front-line practitioners subscribes to the predictions of the Job-Demands Reward model (Siegrist, 1998), thereby providing an avenue of explanation to the unusually high rates of professional turnover (Weinberg and Banks, 2019). This alone should encourage mindfulness in policymakers and administrative authorities of clinical settings when re-engineering the workplace for the sake of cost efficiency. Taking Ontario as an example, the 2000’s saw the end of discipline-based departments in hospitals, leaving many clinical social workers with an “isolated practice and diminished job security” (Aronson and Sammon, 2000: 170). Aronson and Sammon (2000), more than two decades ago, warned of the deleterious effects such restructuring had on the practice and well-being of social workers. From a representational analysis perspective, such a fracture in the professional framework threatens the lived experience of social workers. Their representation of clinical practice is objectified in a crisis management situation that does not allow for the attribution of a durable meaning within the values anchored at the heart of the professional representation. Instability is thus the field of reference for this professional group.

In this high-pressure environment then, the obligatory documentation of practices further destabilizes front-line professionals by imposing an unnatural shift from client-centred priorities to managerial ones (Phillips, 2019), further departing from an authentic actualization of their professional self-representation (Lévesque et al., 2019). The clinical social work practice thereby incurs undue financial responsibilities (Chow et al., 2019) as healthcare social workers advocate their contribution through statistical monitoring. Their lived experience is consequentially burdened by mandatory bureaucracy, increasing their overall work demands with performance metrics ranging from time spent per patient, quantity of notes entered (for use by interdisciplinary team members), type of service offered, number of family meetings coordinated, and weekly ratios of discharges and patients met. Said demands are alleged to be unfairly valued by the administration, whose statistical tools are unsuited to account for the emotionally draining nature of the task as they favour evidence-based practice more readily than the relational and humanistic expertise of clinical social workers. The alarming ratios of compassion fatigue (Thomas, 2013), moral distress (Jessen, 2015) and depression (Siebert, 2004) in healthcare social workers attest to the importance of considering not only the extent of a given workload, but its inherent challenges together. Where NPM reforms have purportedly translated into heightened work precarity for social workers (Baines, 2004), it leaves them reportedly forced to juggle between witnessing the hardships of their clients, the first victims of public retrenchments, and enduring their own sense of precariousness as under-recognized members of the team. This conflictual experience in front-line practitioners is one well-documented by Lipsky and Hill (1993), whose “street-level bureaucrats” come to incarnate the policies that they actualize. It should be a cause for concern that a persisting sense of inequity deriving from unrepresentative performance metrics could become an integral part of the social representation of the practice, anchoring these negative feelings to the representation of the profession itself.

The struggles incurred by the new procedural expectations is worsened by the relationships healthcare social workers have with immediate supervisors and, more importantly, higher management. No guidelines for social-work practice or professional supervision are directly provided, leaving many in a place of confusion with varying standards of practice across their occupational group. These challenges are compounded in practice as clinical social workers are already struggling to achieve internal consistency within their professional identity (Beddoe, 2011; Thyer, 2002). Generally, there exists an understanding that evidence-based practices, along with quantitative metrics, are preferred by the institution (Baines, 2006). However, as accounting-based forms of service provision displaces the central “caring” component of the social work profession, no satisfying alternative has been provided, leaving clinical social workers in an unpleasant practical and representational limbo (Baines, 2006). More conflictual still, the broad managerial demands made to our participants were said to align with differing finalities of practice, as cost-efficiency is prioritized over patient well-being. The lived experience of clinical social workers is therefore strained by the competing ideas of practice held between them and their employers, thus impeding the harmonious objectification and anchoring of the practice into a cohesive professional representation.

As eloquently described by Abramovitz and Zelnick (2018), the managerialism of the workplace leaves little place for social justice, community and compassion by branding social work itself as a form of business within an agency. Our findings align with those of Abramovitz and Zelnick (2018) by attesting to the self-reliance of clinical social workers who “make do” without appropriate support, as funding is unavailable to promote organizational resilience in the form of external training. Further still, our results allow us to reframe the significance of these hierarchical tensions as greater than a simple disagreement in occupational priorities. NPM principles, in essence, contradict the professional representation of social work at its core (Lévesque et al., 2019), thus delegitimizing in the eyes of its professionals those who reify its implementation (i.e., management). Clinical social workers’ very authority figures therefore wield a contested power, leaving each group in continued conflict when cooperation is nonetheless mandated. By exploring our participants’ shared experience, we find that this very dissonance is the source of considerable pain, one with no immediate remedy.

True to the managerial spirit of neoliberal institutions (Abramovitz and Zelnick, 2018), the workplace was found to foster self-sacrifice in front-line practitioners who allege lacking a sufficient recognition for their contribution. Social workers end up sacrificing their well-being in and outside their workplace in order to prove their value, for example by skipping breaks, by working additional hours from home or by paying out of pocket to provide for a patient’s basic needs. While this undue burden to social workers is well documented in the literature (Ben-Zur and Michael, 2007; Bouterfas et al., 2016), our findings contribute to this debate by pointing to the considerable disconnect between NPM reforms and the front-line practitioners’ professional representation, which in turn (de)legitimizes the power of the institution and informs the compensatory strategies deemed acceptable by the group. Indeed, as detailed by Jodelet (2015), the normative background organizes the context wherein individuals endorse social representations. Given the potency of neoliberal values within the healthcare social workers’ place of work, mobilizing individual recourse to palliate the patients’ needs is the only option available to honour one’s professional values without directly opposing the authorities in place. Consciously or not, this bid for recognition benefits the system in that social workers, at their own expense, do more than they should and provide services with limited resources, thereby cutting costs for the institutions and government agencies that employ them.

The distress incurred by predictable changes under a neoliberal agenda points to the professional representation of clinical social workers remaining decidedly critical and resisting the new status quo. Social workers have long contested the neoliberal agenda and advocated for the preservation of the Welfare state (Savignat, 2009; Harris, 2014; Gray et al., 2015; Strier, 2019). As neoliberalism poses a direct threat to the humanistic principles of front-lines practitioners (Payne, 2011), academics and front-line workers alike have rallied to condemn this ideology’s wide-ranging disturbances. Similar to Hyslop (2018), we find that the pain and discontent experienced by clinical social workers in their place of work relate directly to the rejection of the expectations of practice compelled by their institution. Where their contentious lived experience is a result of NPM-informed policies, their professional representation has remained stable even when increasingly incompatible with the context at hand. Indeed, to alleviate the distress caused by this dissonance, clinical social workers are called upon to welcome a core transformation to their professional identity by re-objectifying and re-anchoring their representation of their practice in alignment with the NPM perspective guiding administrative priorities. The cost to healthcare social workers was here found to be unconscionable however, as it would negate their very integrity and purpose. In turn, discrete institutional policies are overturned and resisted in practice as clinical social workers favour their professional values ahead of administrative priorities. In Ontario namely, under restrictions likely common across provinces, organisations limit client-eligibilities and privilege easier cases to optimize their efficiency metrics (Aronson and Smith, 2010). Social workers, conversely, ally themselves with their service beneficiaries either through rule-bending, internal advocacy or outright refusal of impositions deemed unethical (*Ibid*.). This relative independence of practice, however, comes at an unexpected cost.

### Autonomy: From Friend to Foe

To our surprise, during the interview process, autonomy was identified as one of the most significant elements of a negative workplace experience. Its problematic role comes in direct contradiction with previous research on general well-being in an occupational setting (Papathanassoglou et al., 2012), challenging even primary workplace stress models such as Karasek and Theorell (1990), which identify autonomy as a central feature of workplace well-being. Under the theory of social representations however, we are able to understand how group subjectivities shape the meanings underlying a particular context of practice and, in turn, transform its shared experience.

Four interrelated factors were found to define the experience of autonomy for healthcare social workers and anchor it to their representation of the practice. First, discretionary autonomy is challenging when the profession is poorly understood (Lévesque et al., 2019), with interdisciplinary teams and administrators taking advantage of imprecise role boundaries to task social workers with unrelated work, adding to their already heavy workload. The *value-based representation of professionalism* is here mobilized to protect the integrity of social workers’ identity and its original contribution to a team of medically-oriented colleagues in light of a conflicting representation of practice. From our findings, it is made clear that the shared professional representation of social workers remains objectified as value-centered in the face of increasing pressures to adopt what Baines (2006) describes as “accounting-based forms of public sector management” bolstered by “metrics, such as best practices, competencies, and benchmarking” (p.196), in itself a demand which has resulted in a decrease in the quality of social services as a whole (*Ibid.*).

Second, within the lived experience of clinical social workers, autonomy aggravates their vulnerability to litigation by increasing their accountability, as they are required to take on more responsibilities. Through an overview of the *exposure to litigation* element, it becomes clear that social workers’ role and judgment are formally questioned in the legal process, thus reinforcing the self-doubt already triggered by the undervaluation of their practical skills and lack of professional social-work guidelines from administrators. As such, lawsuits serve to further undermine front-line practitioners’ path to recognition, as every aspect of their practice is individually questioned before they may move forward as an occupational entity. This vulnerability is experienced as a threat to the integrity of their professional identity and the legitimacy of their contribution, further reinforcing within the clinical social workers’ representation of their practice the tensions outlined above between clinical social workers and management.

In third, an authentic and complete autonomy is not granted to social workers; instead, they constantly struggle to reconcile the expectations of higher management with their own representation of the social practice. This conditional quality is key, as it deprives social workers of the benefits of a discretionary autonomy which would otherwise allow front-line practitioners to conduct themselves in alignment with the values they hold dear as professionals. The *primacy of field experience* was voiced as an effective means to mitigate these conflicts, thus anchoring a recognizable social work imperative (first-hand intervention knowledge) to answer for a challenging practical reality. Through honing their skills, setting a precedent for practice and being prepared for inevitable litigations, experienced social workers could allay the negative impacts of their uneasy autonomy. That being said, the inner tensions experienced between the representation of the practice and their professional identity never ceased to weigh on our participants, regardless of their seniority.

Finally, the lack of clear professional guidelines and support from management speak once more of the tensions within the representation of the social practice and strips the social workers’ discretionary autonomy of its advantages by only exacerbating the obstacles inherent to their lived experience. This need for true and full autonomy is further rooted in professional tradition as, before the rise of “managed care”, autonomy in North American social workers was unquestionably valued as it ensured one’s “professional responsibility and creativity” (Munson, 1996: 250). In an increasingly litigious workplace with an ever complexifying client-care however, a lack of professional guidance grounded in social work values and, by extension, limited support from one’s employer, leaves front-line practitioners further “out-of-place” and vulnerable in a workplace driven by managerialism and standardized practices fundamentally at odds with the profession. Our findings parallel a previous research on workers’ professional suffering, where New Public Management philosophies led workers to adopt dysfunctional behaviours (Negura and Maranda, 2013). In this study, drug consumption was used as a coping strategy to meet impossible standards of practice set in a context of higher autonomy, where the individual assumes all responsibilities related to their work without access to the necessary resources. Along with ours, this research suggests the unsustainable nature of Canadian neoliberal reforms in a healthcare setting for front-line practitioners and points to a flawed ideology of social service provision at a political level.

It should be stated that some contributors to NPM research have made a compelling argument to move past the opposition between professional groups and NPM-inspired reforms already heavily documented in the literature in favor of exploring the constructive adaptions to one’s professional model that would support a given occupational group under the contemporary reforms of the public sector, a position in direct contrast with the optics of our discussion. Bezes et al. (2012), namely, have argued the interest of debating beyond such opposition in order to better appreciate the ongoing workplace transformations that have successfully transcended this dichotomy. The authors identify promising avenues of research by exploring adaptations on each end of the spectrum (both on the front lines and behind parliament doors) to best serve the interest of the public and to stimulate renewal in professional roles involved with public service provisions. This inclination is founded in Lipsky and Hill (1993) extensive research on street-level bureaucrats and the mutual influence held by policymakers and those who implement them. The benefits of the analytical progression suggested by Bezes et al. (2012) are immediately tangible as they serve to dispel the fear of neo-managerial policy interference on professional identities and practice by pointing to the transformation imperative faced by any professional role over time under the gradual implementation of structural and relational workplace changes. While certainly helpful in the right context, we would argue that this interpretational lens is not suited to the current predicament of Canadian clinical social workers. For one, the professional identity of social workers needs to be better acknowledged and clearly defined internally before welcoming external changes more conciliatory with the institutional ideal (Lévesque et al., 2019). While professions are more than static “statuses”, they require consistency through recognized boundaries before being amenable to transformations incurred from external circumstances. This explains, in turn, why clinical social workers endure greater psychological distress in the workplace over enacting changes to their professional perspective as it has yet to reach a place of stability conducive to such a step (Beddoe, 2011; Thyer, 2002). Moreover, as a consequence of their deontology and training, North American social workers are compelled to act as agents of social change within a humanistic approach to practice (Payne, 2011), in itself a contradiction to the pro-normative reforms inspired by the NPM approach (Abramovitz and Zelnick, 2018). There are limited concessions to be made on either front, therefore, as the transformations requested by each side target the immutable of the other: the values that make them what they are. Finally, while such a point goes far beyond the scope of this article, it should be mentioned in passing that Canadian social workers bear the weight of a controversial history as governmental agents tasked with implementing now widely-recognized condemnable policies targeting marginalized groups (the sixties scoop, to name but one example, which removed first nations’ children from their families) (Blackstock, 2009). Canadian social workers experience daily the stigma associated with their professional reputation (Lévesque et al., 2019) and are thus wary of immediately welcoming any top-down changes in practice.

In sum, our findings deepen our understanding of the social workers’ opposition to NPM reforms by discussing the representational elements of their lived experience along with the processes that maintain their professional continuity even if contributing to their psychological distress in the workplace. Such a critical reflection is necessary to unravel the previously unexplored dynamics at play in the resistance of front-line practitioners to neo-managerial occupational changes. Our article’s aim is to ensure the matters of meaning within this debate are fully examined, thereby giving social workers the tools to navigate future transformations to their professional identity in a manner consistent with their values and mission. This reflexive approach is characteristic of contemporary social workers and aligns with their occupational priorities (Heron, 2005).

### Recommendations for Our Current Social and Workplace Settings

The Covid-19 pandemic has only added to the daily difficulties experienced by clinical social workers. While the current health crisis leaves them at heightened risks of psychological stress and vicarious trauma (Wu et al., 2020), the added burden brought on by our healthcare institutions’ *modus operandi* risk pushing front-line practitioners to their breaking point. Our findings on the lived experience of clinical social workers are all the more relevant that provincial governments are now forced to confront the blatant systemic flaws resulting from decades of erosion to the Welfare State. In effect, the pandemic has exposed the need for rapid and far-ranging changes in policy and institutional functioning. In this time of societal transformation, the well-being of our healthcare workers deserves to be properly acknowledged.

In light of our results, we would recommend four main avenues for improving the lived experience of clinical social workers and, by extension, facilitating their service provision as a whole. For a start, clearer practical guidelines, consistent with social work values, provided by healthcare institutions would not only serve to anchor clinical social workers under coherent expectations of practice, but it could also offer protection from litigation and dispel the disadvantages raised as inherent to their discretionary autonomy. Indeed, if clinical practice conforms to the employers’ protocols, the social workers themselves are absolved from blame for following procedure. Provided these standards are established with respect to the values and priorities of clinical social workers (see Lévesque et al., 2019 for greater details), it could serve to free the occupation from undue fears of prosecution over standard practices. This conforms to recommendations made by Munro (2004) toward the management of social work within the United-Kingdom, whereby it is argued that the profession would benefit from improving its knowledge-base under “more reliable and valid measures of performance” (p. 1093) without depriving social workers from the individual discretion needed to offer a personalized service. More nuanced vision of performance would further work to dispel the defensiveness toward practice monitoring and in contrast bolster clinical social workers’ claim to greater recognition within the workplace. Such administrative targets ought to be handled with care, not only in acknowledgement of the social workers’ needs, but also in light of their litigious workplace context. Indeed, performance metrics wielded by management to further control the practice, as opposed to supporting it, risk incurring entirely new dysfunctional outcomes or even gaming of the data produced to defend against increasing bureaucratic pressures (Hood, 2002; Bevan and Hood, 2006). As is established both within our findings and elsewhere in the literature, social workers conceive of their data entry as the last line of defense against budgetary cuts (Baines, 2006). As such, broad and uninformed additions to monitoring standards risk inciting counteracting behaviors adverse to service quality and workplace well-being, with “ratchet effects, threshold effects and output distortions” (Bevan and Hood, 2006: 521) being but a few examples. No one solution is perfect nor sufficient, therefore, and should result from an active collaboration with the occupational group that it concerns.

Our findings further call for greater resources to be made generally available to healthcare social workers. Namely, better staffing along with stronger and more varied public services would help decrease front-line practitioners’ caseload and afford them the time to properly counsel and support their service beneficiaries under a humanistic frame of practice. Third, beyond pragmatic considerations of the workplace experience, improvements are warranted to support clinical social workers’ self-worth and sense of value within the institution. Significant pain is experienced from our participants perceptions of disregard. This distress could be alleviated namely by allowing some healthcare social workers to achieve managerial positions and relate to the front-line practitioners that they once worked alongside with. Moreover, it would be crucial to ensure that interdisciplinary colleagues and authority figures alike are well-aware of the professional ethos of social workers for their expectations to not overstep their employees’ occupational boundaries. Fourth, consulting clinical social workers ahead of institutional retrenchments would also come a long way to make them feel heard and, at the very least, enable them to advocate for the importance of their work in a meaningful way outside constant notetaking. In sum, we offer these recommendations in service to healthcare institutions seeking to ensure the strength and continued engagement of their clinical social workers, particularly in these trying times.

### Limitations of Our Study

Having elaborated on our findings, it is important to address the limitations of our study and how they may have impacted our results. As participants were required to self-identify as having recently experienced distress in the workplace, it is possible that their reactions to organizational constraints and their perceived significance could have been heightened by a compromised psychological disposition or a previously vulnerable state of mind. All appropriate measures were taken, however, to ensure an adequate state of mind from our participants during the interview. The interview questions were additionally formulated in order to promote discussions on experiences applicable to themselves and their occupational group more broadly in hopes of limiting this personal bias. Given the inductive nature of our approach, our methodology is also not immune from researcher bias. While the data was reviewed by both authors independently and organized in terms of significance through frequency and occurrence, prior expectations could have influenced our understanding of the results.

It should be mentioned that our appreciation of NPM reforms in Canadian healthcare institutions has focused on their practical consequences as observed by our participants and corroborated by the literature. While the neoliberal ideology has rooted decades of healthcare policies (Bart and Hupfer, 2004), their resulting implementation in the workplace varies largely according to the provincial ministries at hand and the institutional administrators tasked with its management. It ought to be said that this article’s aim is not to provide a neutral portrait of the specific policies to be blamed for the occupational obstacles facing clinical social workers today. Rather, we sought to explore the latter’s own understanding of institutional praxes informed by NPM priorities, substantiated for the most part by the relevant literature on the professional reality of social workers.

We also recognize that our data is not wholly representative of all Canadian social workers in a healthcare setting, given the eligibility criteria for participation (female, francophone and from urban centres only). Further, our recruitment efforts yielded a majority of Caucasian participants with a limited representation of other cultural or ethnic groups than French-Canadian. We have no way of determining at this time how this restricted diversity could affect our results. That said, as our sample was recruited from three distinct provinces through a number of healthcare institutions, we argue that our study still offers generalizable results. Indeed, the organisational context remains stable across provinces regardless of linguistic affiliation, gender or individual cultural heritage. We hence contend that this exploratory study provides fruitful avenues of reflection about social workers’ experience of their professional environment and remains of value to inform future research on front-line practitioners’ workplace lived experience and the impacts of healthcare reforms on the quality of social services.

## Conclusion

Our findings attest to the variety of challenges experienced by social workers following the last decade of healthcare reforms in Canada. Front-line practitioners endure numerous organizational constraints which reorient their practice in a direction which does not align with their sensibilities, priorities and values. In this context, the “paradoxical autonomy” resulting from these reforms becomes an unwelcome reality of their work environment, as it masks deficient guidelines and supervision under the guise of discretionary practice. From these results, we would argue that the lived experience of the workplace by healthcare social workers is shaped by their multifaceted representation of practice and the inherent dynamics at odds with a neoliberal workplace context. Indeed, the conflicts in practice, value and identity maintained by representational dissensions negatively influence the work lives of clinical social workers.

Within the context of the greater research project under which this study has been conceived (see Negura and Lévesque, 2021 for reference), these findings uniquely contribute to our understanding of professional distress by relating it to a lived experience of institutional constraints and expectations. Specific to this article are the ties uncovered between the common responses to given occupational circumstances, the sensitivities they generate and the professional representation that explain them. Outside our broader investigative efforts, a representational analysis of the lived experience of healthcare social workers further nuances the debate surrounding the impact of NPM reforms on social work beyond its immediately observable consequences, such as the bureaucratization of the practice (Burton and van den Broek, 2009), the greater push for crisis management or the rising workload (Abramovitz and Zelnick, 2018; Aronson and Sammon, 2000). The shared distress experienced by clinical social workers is here discussed as not only a matter of circumstances (either psychological or social in nature), but also as unresolved tensions within the professional ethos. As neoliberal imperatives further disconnect clinical social workers from their humanistic endeavours in favor of efficiency and measurable outputs, the very significance of the occupation is voided. In other words, we present an understanding of workplace distress as an affliction of meaning, one which plays a determining role in the nature of the psychological stress experienced and highlights the social ramifications of broader healthcare decision. Nowhere is this clearer than in the face of our findings on the social workers’ autonomy of practice. As a negative determinant of workplace well-being, our results contradict most common psychological models of work-related stress (Karasek and Theorell, 1990; Siegrist, 1998). Under a representational lens however, our findings are congruently explained in light of the persisting tensions between social workers and their management along with the power dynamics that subsumes them and the strategies adopted to maintain the occupational group’s representational continuity.

With clear empirical evidence of the undue stress experienced by healthcare social workers (Evans et al., 2005), this research may serve to assist policy makers and administrators to rethink healthcare reforms beyond the aims of financial efficacy and individualised care, but also in terms of their feasibility for care providers. Workplace well-being initiatives may equally gain from prioritizing changes at the organizational level, thus improving social workers’ experience by alleviating both pragmatic and representational drivers of their occupational distress. Finally, better supported practitioners would directly entail better patient and family services, as social workers would be enabled in their work.

## Data Availability

The raw data supporting the conclusions of this article will be made available by the authors, without undue reservation.
